# Supplementary material for: Gender-Related Patterns of Emotion Regulation among Patients with Eating Disorders
Source: J Clin Med. 2019 Feb 1;8(2):161. doi: 10.3390/jcm8020161 (PMC6406611; doi:10.3390/jcm8020161)
Supplement: Supplementary file 1 [file jcm-08-00161-s001.pdf]

Journal of
Clinical Medicine

MDPI

Table S1 (supplementary material). SEM: tests of direct, indirect and total effects (ED subsample, *n*=718).

Direct effects		Coef.	Std. Err.	z	P> z	Std. Coef.
DERS	SCL_GSI	2.780192	.2584709	10.76	0.000	.416584
	EDI_TO	.0262036	.0045259	5.79	0.000	.2339745
	TCI	2.772943	.3770577	7.35	0.000	.3323533
	Age	-.0238836	.0093216	-2.56	0.010	-.0452157
	Sex	-.2372371	.2906999	-0.82	0.414	-.0143486
TCI	Sex	-.271952	.0651474	-4.17	0.000	-.137234
NS	TCI	3.500691	.7668622	4.56	0.000	.1492974
HA	TCI	22.9205	.9150037	25.05	0.000	.7244588
RD	TCI	-5.11636	.7846171	-6.52	0.000	-.2202577
PS	TCI	-6.817563	1.025106	-6.65	0.000	-.2251357
SD	TCI	-30.85276	1.042722	-29.59	0.000	-.89173
CO	TCI	-7.216501	.7558629	-9.55	0.000	-.3160973
ST	TCI	3.534692	.7369039	4.80	0.000	.1531579
Non-accep	DERS	1	(constrained)			.7614167
Goals	DERS	.6784324	.0281205	24.13	0.000	.7242732
Impulse	DERS	.9080089	.0345015	26.32	0.000	.7801191
Aware	DERS	.2853168	.0289913	9.84	0.000	.3145658
Strategy	DERS	1.439858	.0425057	33.87	0.000	.8977073
Clarity	DERS	.6432919	.0278365	23.11	0.000	.701024
SCL_GSI	TCI	1	(constrained)			.7998917
	Age	.0085171	.0015578	5.47	0.000	.10761
	Sex	-.2364237	.0497222	-4.75	0.000	-.0954315
EDI_TO	TCI	60.92764	1.418891	42.94	0.000	.8178347
	Age	.3499298	.0897122	3.90	0.000	.0741931
	Sex	-16.28712	2.868973	-5.68	0.000	-.1103227

Indirect effects		Coef.	Std. Err.	z	P> z	Std. Coef.
DERS	TCI	4.376714	.3381154	12.94	0.000	.5245745
	Age	.0328485	.0065788	4.99	0.000	.0621879
	Sex	-3.028448	.474595	-6.38	0.000	-.1831676
NS	Sex	-.9520201	.3047151	-3.12	0.002	-.0204887
HA	Sex	-6.233278	1.496592	-4.16	0.000	-.0994204
RD	Sex	1.391405	.3825685	3.64	0.000	.0302268
PS	Sex	1.85405	.513825	3.61	0.000	.0308963
SD	Sex	8.39047	1.985628	4.23	0.000	.1223757
CO	Sex	1.962542	.4948809	3.97	0.000	.0433793
ST	Sex	-.9612667	.3041239	-3.16	0.002	-.0210185
DERS_No	SCL_GSI	2.780192	.2584709	10.76	0.000	.3171941
	EDI_TO	.0262036	.0045259	5.79	0.000	.1781521
	TCI	7.149657	.2454397	29.13	0.000	.6524792
	Age	.0089649	.011533	0.78	0.437	.0129229
	Sex	-3.265685	.5383351	-6.07	0.000	-.1503922
DERS_Go	SCL_GSI	1.886172	.1755035	10.75	0.000	.3017207
	EDI_TO	.0177774	.0030405	5.85	0.000	.1694614
	TCI	4.850559	.184782	26.25	0.000	.6206499
	Age	.0060821	.0078129	0.78	0.436	.0122925
	Sex	-2.215546	.3660403	-6.05	0.000	-.1430557
DERS_Im	SCL_GSI	2.524439	.2329607	10.84	0.000	.3249852
	EDI_TO	.0237931	.0040704	5.85	0.000	.1825279
	TCI	6.491952	.2184681	29.72	0.000	.6685058
	Age	.0081402	.0104651	0.78	0.437	.0132403
	Sex	-2.965271	.4876699	-6.08	0.000	-.1540862
DERS_Aw	SCLgsi	.7932356	.1020997	7.77	0.000	.1310431
	EDI_TO	.0074763	.0014945	5.00	0.000	.0736004
	TCI	2.039917	.2049221	9.95	0.000	.2695602
	Age	.0025578	.0032949	0.78	0.438	.0053389
	Sex	-.9317549	.176609	-5.28	0.000	-.0621318
DERS_St	SCLgsi	4.003083	.3584175	11.17	0.000	.3739705
	EDI_TO	.0377294	.0064274	5.87	0.000	.2100406
	TCI	10.29449	.2752929	37.39	0.000	.7692703
	Age	.0129082	.016591	0.78	0.437	.015236
	Sex	-4.702124	.7667856	-6.13	0.000	-.1773117
DERS_Cl	SCLgsi	1.788475	.1655937	10.80	0.000	.2920354
	EDI_TO	.0168565	.0029442	5.73	0.000	.1640217
	TCI	4.599317	.1847327	24.90	0.000	.6007269
	Age	.0057671	.0074081	0.78	0.436	.0118979

	Sex	-2.100789	.3484663	-6.03	0.000	-.1384636
SCL_GSI	Sex	-.271952	.0651474	-4.17	0.000	-.1097723
EDI_TO	Sex	-16.5694	3.96271	-4.18	0.000	-.1122347

**Total effects**		**Coef.**	**Std. Err.**	**z**	**P> z**	**Std. Coef.**
DERS	SCL_GSI	2.780192	.2584709	10.76	0.000	.416584
	EDI_TO	.0262036	.0045259	5.79	0.000	.2339745
	TCI	7.149657	.2454397	29.13	0.000	.8569278
	Age	.0089649	.011533	0.78	0.437	.0169722
	Sex	-3.265685	.5383351	-6.07	0.000	-.1975162
TCI	Sex	-.271952	.0651474	-4.17	0.000	-.137234
TCI_NS	TCI	3.500691	.7668622	4.56	0.000	.1492974
	Sex	-.9520201	.3047151	-3.12	0.002	-.0204887
TCI_HA	TCI	22.9205	.9150037	25.05	0.000	.7244588
	Sex	-6.233278	1.496592	-4.16	0.000	-.0994204
TCI_RD	TCI	-5.11636	.7846171	-6.52	0.000	-.2202577
	Sex	1.391405	.3825685	3.64	0.000	.0302268
TCI_PS	TCI	-6.817563	1.0251			
06	-6.65	0.000	-.2251357			
	Sex	1.85405	.513825	3.61	0.000	.0308963
TCI_SD	TCI	-30.85276	1.042722	-29.59	0.000	-.89173
	Sex	8.39047	1.985628	4.23	0.000	.1223757
TCI_CO	TCI	-7.216501	.7558629	-9.55	0.000	-.3160973
	Sex	1.962542	.4948809	3.97	0.000	.0433793
TCI_ST	TCI	3.534692	.7369039	4.80	0.000	.1531579
	Sex	-.9612667	.3041239	-3.16	0.002	-.0210185
DERS_No	SCL_GSI	2.780192	.2584709	10.76	0.000	.3171941
	EDI_TO	.0262036	.0045259	5.79	0.000	.1781521
	DERS	1	(constrained)			.7614167
	TCI	7.149657	.2454397	29.13	0.000	.6524792
	Age	.0089649	.011533	0.78	0.437	.0129229
	Sex	-3.265685	.5383351	-6.07	0.000	-.1503922
DERS_Go	SCL_GSI	1.886172	.1755035	10.75	0.000	.3017207
	EDI_TO	.0177774	.0030405	5.85	0.000	.1694614
	DERS	.6784324	.0281205	24.13	0.000	.7242732
	TCI	4.850559	.184782	26.25	0.000	.6206499
	Age	.0060821	.0078129	0.78	0.436	.0122925
	Sex	-2.215546	.3660403	-6.05	0.000	-.1430557
DERS_Im	SCL_GSI	2.524439	.2329607	10.84	0.000	.3249852
	EDI_TO	.0237931	.0040704	5.85	0.000	.1825279
	DERS	.9080089	.0345015	26.32	0.000	.7801191
	TCI	6.491952	.2184681	29.72	0.000	.6685058
	Age	.0081402	.0104651	0.78	0.437	.0132403
	Sex	-2.965271	.4876699	-6.08	0.000	-.1540862
DERS_Aw	SCL_GSI	.7932356	.1020997	7.77	0.000	.1310431
	EDI_TO	.0074763	.0014945	5.00	0.000	.0736004
	DERS	.2853168	.0289913	9.84	0.000	.3145658
	TCI	2.039917	.2049221	9.95	0.000	.2695602
	Age	.0025578	.0032949	0.78	0.438	.0053389
	Sex	-.9317549	.176609	-5.28	0.000	-.0621318
DERS_St	SCL_GSI	4.003083	.3584175	11.17	0.000	.3739705
	EDI_TO	.0377294	.0064274	5.87	0.000	.2100406
	DERS	1.439858	.0425057	33.87	0.000	.8977073
	TCI	10.29449	.2752929	37.39	0.000	.7692703
	Age	.0129082	.016591	0.78	0.437	.015236
	Sex	-4.702124	.7667856	-6.13	0.000	-.1773117
DERS_Cl	SCL_GSI	1.788475	.1655937	10.80	0.000	.2920354
	EDI_TO	.0168565	.0029442	5.73	0.000	.1640217
	DERS	.6432919	.0278365	23.11	0.000	.701024
	TCI	4.599317	.1847327	24.90	0.000	.6007269
	Age	.0057671	.0074081	0.78	0.436	.0118979
	Sex	-2.100789	.3484663	-6.03	0.000	-.1384636
SCL_GSI	TCI	1	(constrained)			.7998917
	Age	.0085171	.0015578	5.47	0.000	.10761
	Sex	-.5083757	.0735212	-6.91	0.000	-.2052038
EDI_TO	TCI	60.92764	1.418891	42.94	0.000	.8178347
	Age	.3499298	.0897122	3.90	0.000	.0741931
	Sex	-32.85651	4.378354	-7.50	0.000	-.2225574
